# Immunohistochemical Characterization of a Large Cohort of Triple Negative Breast Cancer

**DOI:** 10.1177/10668969231171936

**Published:** 2023-06-12

**Authors:** Rachel Han, Sharon Nofech-Mozes, Dina Boles, Hannah Wu, Nikolina Curcin, Elzbieta Slodkowska

**Affiliations:** 1 Department of Laboratory Medicine and Pathobiology, 7938 University of Toronto, Toronto, Canada; 2 Department of Laboratory Medicine and Molecular Diagnostics, 71545 Sunnybrook Health Sciences Centre, Toronto, Canada; 3 Department of Laboratory Medicine and Pathology, 60444 Markham Stouffville Hospital, Markham, Canada; 4 Department of Pathology, 25478 Southlake Regional Health Centre, Newmarket, Canada; 5 Department of Pathology, 60407 William Osler Health System, Brampton, Canada

**Keywords:** triple negative breast cancer, immunohistochemistry, GATA3, mammaglobin, SOX10, apocrine, antibody clones

## Abstract

*Introduction.* Triple negative breast carcinomas are characterized by a lack of hormone receptor and HER2 expression and inconsistent expression of breast-specific immunohistochemical markers. The expression of many site-specific markers in these tumors is largely unknown. The objective of the study was to examine the expression of widely used immunohistochemical markers on a large cohort of triple negative breast cancer. *Methods.* Sections from tissue microarrays were stained with 47 markers using routine protocols. Most markers were scored using a modified Allred method. ATRX, BAP1, SMAD4, e-cadherin, and beta-catenin were scored as retained or lost. Mammaglobin was considered positive if there was at least moderate intensity staining in any tumor cells. P16 was scored as overexpressed or not overexpressed; p53 was scored as wildtype, overexpressed, null, or cytoplasmic. *Results.* The cohort consisted of 639 tumors including 601 primary and 32 metastases. Overall, 96% expressed GATA3, mammaglobin, and/or SOX10 while 97% of no special type tumors expressed this panel. Carcinoma of apocrine differentiation demonstrated an AR positive, SOX10 negative, K5 negative/focal immunophenotype. PAX8 (SP348), WT1, Napsin A, and TTF1 (8G7G3/1) were never or rarely expressed while CA9, CDX2, NKX3.1, SATB2 (SATBA410), synaptophysin, and vimentin were variably expressed. *Conclusions.* Almost all TNBC express at least 1 of the 3 IHC markers: GATA3, mammaglobin, and/or SOX10. Carcinoma of apocrine differentiation is characterized by an AR positive, SOX10 negative, K5 negative or focal immunophenotype. Cautious interpretation of so-called site-specific markers, with knowledge of antibody clones, is required in excluding the diagnosis of triple negative breast cancer.

## Introduction

Triple negative breast cancers (TNBCs) are a pathologically and clinically heterogenous group of carcinomas defined by a lack of expression for estrogen receptor, progesterone receptor and human epidermal growth factor receptor 2 (HER2).^1,2^ Their proclivity for metastases notwithstanding, the diagnosis of TNBC in the metastatic setting is inherently challenging, owing to the lack of staining for hormone receptors and many breast-specific immunohistochemical (IHC) markers.

Studies evaluating GATA-binding protein 3 (GATA3), one of the most widely employed breast-specific IHC markers, have demonstrated decreased levels of expression among TNBCs.^3‐7^ Similarly, the expression of mammaglobin and gross cystic disease fluid protein 15 (GCDFP15) have been demonstrated to be significantly lower in TNBCs than in hormone receptor positive breast cancers.^7‐11^ More recently, Sry-related high-mobility-group/HMG box 10 (SOX10) has emerged as a potential marker with increased sensitivity and specificity for the diagnosis of TNBC; however, reported expression rates vary widely in the literature, ranging from 38% to 67%.^12‐17^

Though the distinction of TNBC in both the primary and metastatic setting carries significant therapeutic and prognostic implications, a specific, reproducible immunophenotype for diagnosing these tumors has not yet been well defined. Further, there is limited data on the expression of many site- or disease-specific IHC markers in TNBC. Therefore, the purpose of this study was to examine the expression of widely available IHC markers on a large cohort of TNBC as it pertains to the daily practice of pathology.

## Materials and Methods

Research and Ethics Board approval was obtained from all participating institutions. TNBC diagnosed between 2010 and 2019 were identified from the Departments of Pathology at Sunnybrook Health Sciences Centre (Toronto), Southlake Regional Health Centre (Newmarket), Markham Stouffville Hospital (Markham) and William Osler Health System (Brampton). Inclusion criteria included tumor size of at least 0.5 cm on excision specimens and adequate tumor cellularity in post-neoadjuvant therapy specimens for tissue microarrays (TMA) construction. Rare tumors (accounting for less than 5% of the cohort) with ER and/or PR expression less than 10% were also included.

All tissues for this study were fixed and processed as per current standards validated for clinical practice. Cold ischemic time and fixation time for most tissues met the recommendations of the current ASCO/CAP guidelines and while they were not available for a subset of tumors, based on review of a representative H&E from each tumor all tissues appeared to be of satisfactory quality. None of the tissues underwent decalcification as none involved bone specimens. All IHC protocols for all IHC markers used in the study were optimized and validated for clinical use according to current standards and showed satisfactory performance on control tissues including limit of detection tissues if known. In addition all IHC was performed in a laboratory that constantly participates in proficiency testing from multiple providers (Nordic Immunohistochemical Quality Control, Canadian Pathology Quality Assurance, College of American Pathologists) with satisfactory results.

All tumors were reviewed and classified by type according to the 5th edition of the WHO Classification of Breast Tumors based on a single representative full section H&E tumor slide review combined with pathology report review. Metaplastic carcinomas were further subclassified into low-grade adenosquamous carcinoma, spindle cell carcinoma, squamous cell carcinoma, matrix-producing, or mixed. All metastatic tumors included in this study came from patients with a history of breast cancer.

Representative archival formalin-fixed paraffin blocks were retrieved for the construction of TMA in triplicate (1 mm diameter) cores. Sections from the TMA blocks were cut at 4 μm for IHC. Forty-seven IHC markers were studied (Table 1) including 2 antibody clones for TTF1 (SPT24, 8G7G3/1), PAX8 (SP348, MRQ-50), and SATB2 (EP281, SATBA4B10). Scoring was completed by 2 breast pathologists and 1 resident. There was a minimum of 2 scorers for each tumor. All discrepant scores were reviewed for consensus. For the majority of the IHC markers, scoring was completed using a modified Allred method, wherein the proportion score (0  =  <1%, 1  =  1%-9%, 2  =  10%-24%, 3  =  25%-49%, 4  =  ≥50%) was added to the stain intensity score (1  =  weak, 2  =  moderate, 3  =  strong staining) for each tumor. Any combined score ≥2 was considered positive. The proportion of tumors that demonstrated diffuse positive staining, defined as strong staining in greater than 50% of the tumor (modified Allred score of 7), was also calculated. With respect to ATRX, BAP1, SMAD4, e-cadherin, and beta-catenin, staining was scored as retained (expression in all/almost all tumor cells), or lost; for the latter 2 markers, tumors with partial loss of expression were identified. Mammaglobin was considered positive when cytoplasmic staining of at least moderate intensity was present in any tumor cells. P16 was scored as overexpressed (block-type staining of strong intensity in virtually all tumor cells) or not overexpressed. P53 was scored as wildtype or abnormal (overexpressed, null, or cytoplasmic staining) as previously described.^18^

**Table 1.** Antibody characteristics.

Antibody	Clone/vendor	Dilution	Stainer	Visualization system	Scoring method
ALK	OTI1A4/Origene	1:1000	DAKO Omnis	EnVision Flex	Modified Allred
AR	SP107/Cell Marque	RTU	Benchmark ultra	UltraView	Modified Allred
ATRX	polyclonal/Sigma	1:800	Benchmark ultra	OptiView	Retained vs lost
BAP1	C4/Santa Cruz	1:100	DAKO Omnis	EnVision Flex	Retained vs lost
Beta-catenin	Beta-catenin-1/DAKO	1:2000	Benchmark ultra	OptiView	Retained vs lost
BRAF	VE1/Abcam	1:400	DAKO Omnis	EnVision Flex	Modified Allred
CA9	polyclonal/Novus	1:2000	Benchmark ultra	OptiView	Modified Allred
CD31	JC70A/DAKO	1:200	DAKO Omnis	EnVision Flex	Modified Allred
CD34	QBEnd10/Roche	RTU	Benchmark ultra	UltraView	Modified Allred
CDX2	EPR2764Y/Cell Marque	RTU	Benchmark ultra	UltraView	Modified Allred
Chromogranin	LK2H10/Roche	RTU	Benchmark ultra	UltraView	Modified Allred
E-cadherin	36/Roche	RTU	Benchmark ultra	UltraView	Retained vs lost
EMA	E29/Roche	RTU	Benchmark ultra	UltraView	Modified Allred
ERG	EPR3864/Roche	RTU	Benchmark ultra	OptiView	Modified Allred
GATA3	L50-823/Cell Marque	RTU	Benchmark ultra	OptiView	Modified Allred
HMWK	34BE12/Roche	RTU	Benchmark ultra	UltraView	Modified Allred
K5	SP27/Roche	RTU	Benchmark ultra	OptiView	Modified Allred
K7	SP52/Roche	RTU	Benchmark ultra	UltraView	Modified Allred
LMWK	B22.1/B23.1/Roche	RTU	Benchmark ultra	UltraView	Modified Allred
Mammaglobin	304-1A5/DAKO	1:100	Benchmark ultra	OptiView	Modified Allred
MelanA	A103/Roche	RTU	Benchmark ultra	OptiView	Modified Allred
Melanosome specific antigen (HMB45)	HMB-45/Roche	RTU	Benchmark ultra	UltraView	Modified Allred
MYB	EP769Y/Abcam	1:200	DAKO Omnis	EnVision Flex	Modified Allred
Napsin A	MRQ60/Roche	RTU	Benchmark ultra	OptiView	Modified Allred
NKX3.1	EP356/Roche	RTU	Benchmark ultra	OptiView	Modified Allred
p16	E6H4/Roche	RTU	Benchmark ultra	OptiView	Overexpressed vs non-overexpressed
p53	DO-7/DAKO	RTU	DAKO Omnis	EnVision Flex	Wildtype vs abnormal
p63	4A4/Roche	RTU	Benchmark ultra	UltraView	Modified Allred
Pankeratin	AE1/AE3/Roche	RTU	Benchmark ultra	UltraView	Modified Allred
PAX8	MRQ-50/Cell Marque	RTU	Benchmark ultra	OptiView	Modified Allred
PAX8	SP348/Abcam	1:400	DAKO Omnis	EnVision Flex	Modified Allred
Podoplanin (D240)	D240/Roche	RTU	Benchmark ultra	UltraView	Modified Allred
S100	polyclonal/DAKO	RTU	DAKO Omnis	EnVision Flex	Modified Allred
SATB2	EP281/Cell Marque	1:400	DAKO Omnis	EnVision Flex	Modified Allred
SATB2	SATBA4B10/Santa Cruz	1:200	DAKO Omnis	EnVision Flex	Modified Allred
SMAD4	EP618Y/Abcam	1:2000	DAKO Omnis	EnVision Flex	Retained vs lost
SOX10	A2/Santa Cruz	RTU	Benchmark ultra	OptiView	Modified Allred
Synaptophysin	SP11/Roche	RTU	Benchmark ultra	OptiView	Modified Allred
TTF1	8G7G3/1/DAKO	1:200	Benchmark ultra	OptiView	Modified Allred
TTF1	SPT24/Leica	1:100	Benchmark ultra	UltraView	Modified Allred
Vimentin	V9/Roche	RTU	Benchmark ultra	UltraView	Modified Allred
WT1	6F-H2/Roche	RTU	Benchmark ultra	OptiView	Modified Allred

Abbreviations: RTU: ready to use.

All IHC protocols for this study used heat as the epitope retrieval method except pankeratin (both heat and protease) and CD34 (none). The external positive controls were used as per NordiQC.org recommendations; for IHC markers not expressed in normal tissues, tumor tissues were used as controls.

## Results

**Figure 1.**
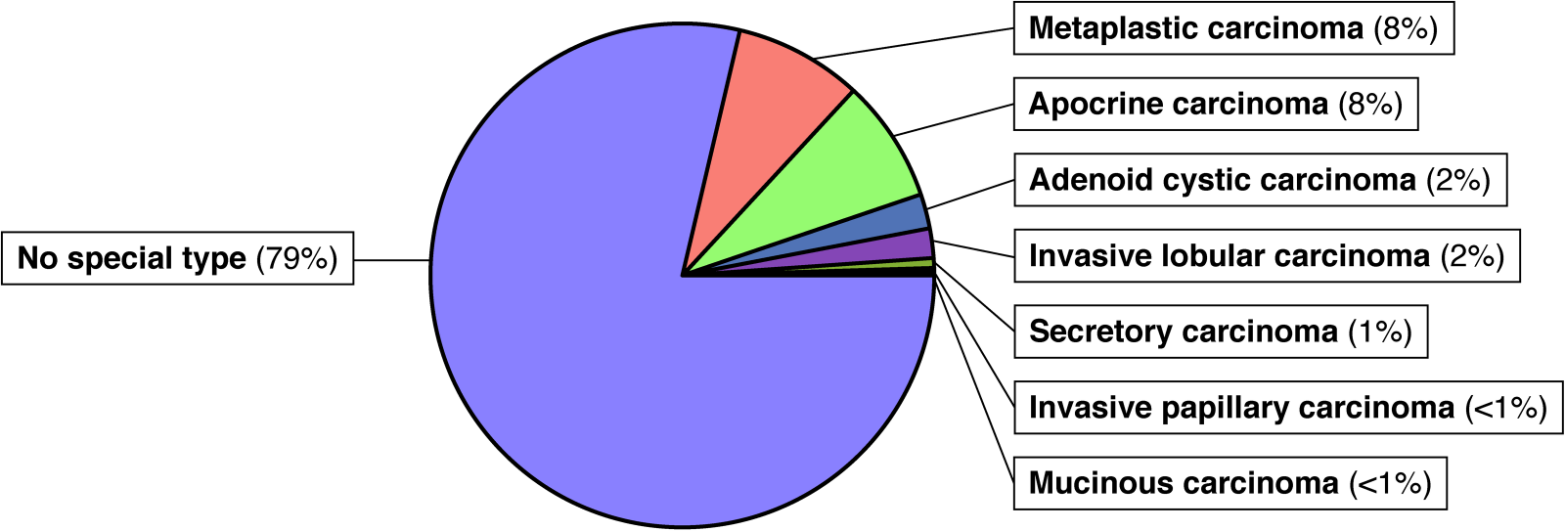
Triple negative breast cancer subtypes represented in our cohort.

The study cohort consisted of 639 TNBC from 628 patients. Tumor sampling included 601 specimens from the breast, 25 from brain metastases, 6 from lymph node metastases, and 1 from a lung metastasis. Two separate primary tumors were included from 4 patients, matched primary and recurrent/metastatic tumors were included from 6 patients, and 2 metastases were included from 1 patient. Seventy-four samples represented post-neoadjuvant therapy specimens. Six specimens were excluded from the study following TMA creation due to a lack of adequate tumor sampling. The resulting study cohort included 499 invasive breast carcinomas of no special type (79%), 53 metaplastic (8%), 50 apocrine (8%), 13 adenoid cystic (2%), 12 invasive lobular (2%), 4 secretory (1%), 1 mucinous (<1%), and 1 invasive papillary carcinoma (<1%) (Figure 1). The results of the IHC studies are presented in Table 2.

**Table 2.** Immunohistochemical marker expression in triple negative breast carcinoma.

IHC marker	Positive	Diffuse positive	Negative
ALK	2% (11/627)	<1% (1/627)	98% (616/627)
AR	38% (242/630)	20% (129/630)	62% (388/630)
CA9	36% (225/623)	4% (23/623)	64% (398/623)
CD31	4% (26/622)	<1% (3/622)	96% (596/622)
CD34	0% (0/622)	0% (0/622)	100% (622/622)
CDX2	9% (54/631)	<1% (2/631)	91% (577/631)
Chromogranin	1% (4/624)	<1% (3/624)	99% (620/624)
EMA	92% (579/627)	67% (423/627)	8% (48/627)
ERG	2% (14/627)	<1% (1/627)	98% (613/627)
GATA3	78% (489/629)	36% (228/629)	22% (140/629)
HMWK	94% (590/631)	64% (401/631)	6% (41/631)
K5	84% (529/628)	58% (367/628)	16% (99/628)
K7	97% (610/631)	91% (572/631)	3% (21/631)
LMWK	97% (608/630)	73% (461/630)	3% (22/630)
Mammaglobin	40% (253/625)	5% (29/625)	60% (372/625)
MelanA	1% (7/630)	0% (0/630)	99% (623/630)
Melanosome specific antigen (HMB45)	1% (6/630)	0% (0/630)	99% (624/630)
MYB	4% (27/622)	0% (0/622)	96% (595/622)
Napsin A	0% (0/628)	0% (0/628)	100% (628/628)
NKX3.1	13% (85/631)	1% (5/631)	87% (546/631)
p63	17% (108/626)	3% (17/626)	83% (518/626)
Pankeratin	99% (622/629)	94% (591/629)	1% (7/629)
PAX8 (MRQ-50)	3% (9/269)	<1% (2/269)	97% (260/269)
PAX8 (SP348)	0% (0/620)	0% (0/620)	100% (621/620)
Podoplanin (D240)	2% (15/630)	<1% (1/630)	98% (615/630)
S100	45% (284/629)	13% (79/629)	55% (345/629)
SATB2 (EP281)	3% (20/626)	<1% (3/626)	97% (606/626)
SATB2 (SATBA4B10)	17% (47/268)	4% (10/268)	83% (221/268)
SOX10	62% (387/629)	54% (341/629)	38% (242/629)
Synaptophysin	11% (67/624)	1% (5/624)	89% (557/624)
TTF1 (8G7G3/1)	<1% (3/627)	<1% (1/627)	>99% (624/627)
TTF1 (SPT24)	1% (9/625)	<1% (2/625)	99% (616/625)
Vimentin	69% (431/628)	31% (193/628)	31% (197/628)
WT1	4% (27/628)	1% (4/628)	96% (601/628)
	**Retained**	**Partial Loss**	**Loss**
ATRX	95% (599/628)	<1% (1/628)	4% (28/628)
BAP1	97% (599/620)	0	3% (21/620)
Beta-catenin	94% (587/626)	3% (20/626)	3% (19/626)
E-cadherin	92% (578/630)	4% (27/630)	4% (25/630)
SMAD4	>99% (624/625)	0	<1% (1/625)
	**Negative/Wildtype**	**Aberrant**	
BRAF	>99% (628/629)	<1% (1/629)	
p16	49% (307/628)	51% (321/628)	
p53	22% (138/627)	78% (489/627)	

**Figure 2.**
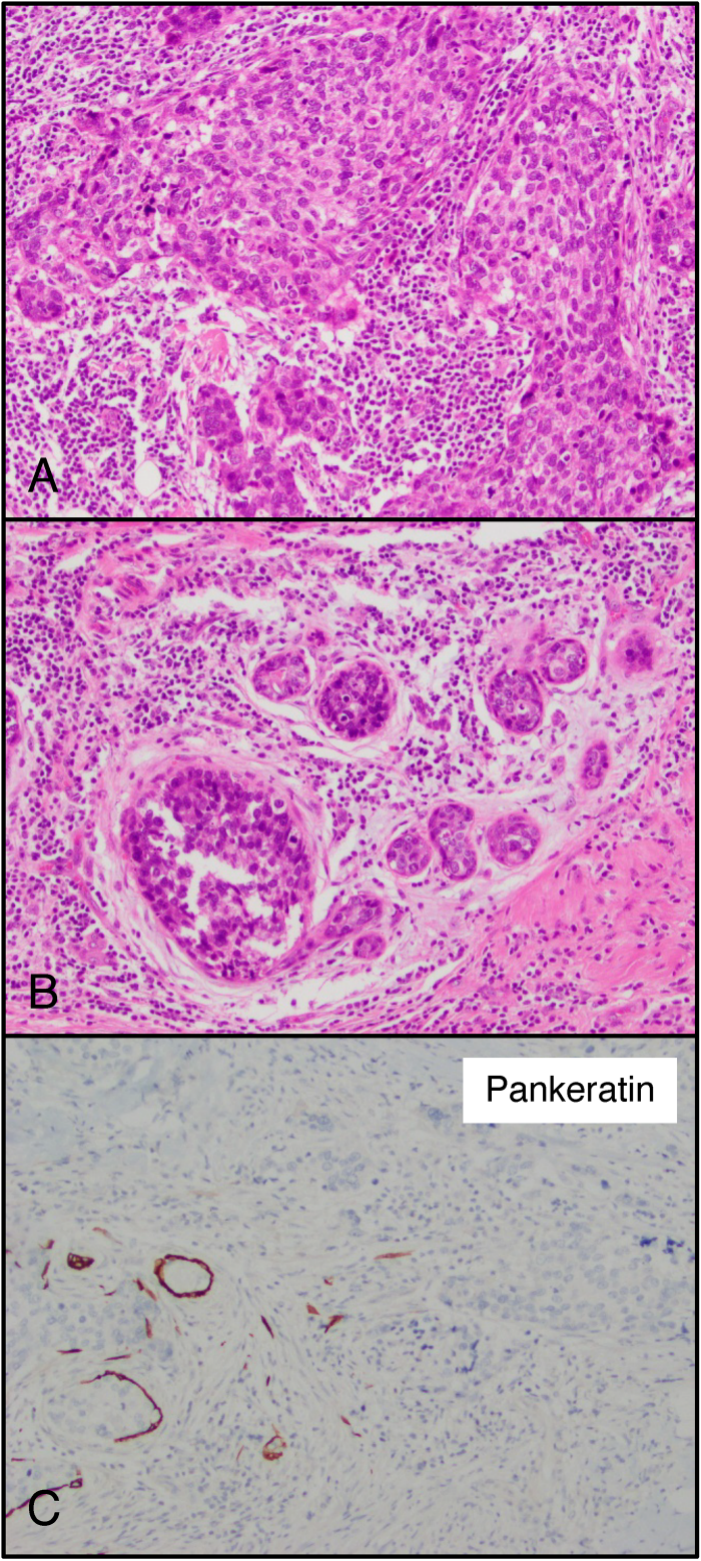
Pankeratin negative triple negative carcinoma of no special type. (A and B) Representative hematoxylin–eosin (H&E) including ductal carcinoma in situ component. (C) Pankeratin, expression in myoepithelial cells and less than 1% of tumor cells (original magnification 20× [A through C]).

Pankeratin was positive in 99% (622 of 629) of tumors. Of the 7 negative tumors, 6 were metaplastic carcinomas (of spindle and matrix-producing subtypes) and 1 was invasive breast carcinoma of no special type (Figure 2). The invasive breast carcinoma of no special type demonstrated an in situ component, positivity for SOX10 and GATA3, and the patient had no other oncologic history. Keratin 7 (K7) was positive in 97% (610 of 631) of tumors. The majority of negative cancers were classified as metaplastic carcinoma (13 of 21) in addition to 7 invasive breast carcinomas of no special type and 1 apocrine carcinoma. Of the 7 invasive breast carcinomas of no special type, 2 were positive for GATA3, 2 were positive for SOX10, and 1 was positive for both GATA3 and SOX10.

P63 was strongly and diffusely positive in 3% (17 of 626) of tumors. Of these tumors, 6 were invasive breast carcinomas of no special type, representing 1% (6 of 493) of all invasive breast carcinomas of no special type.

**Figure 3.**
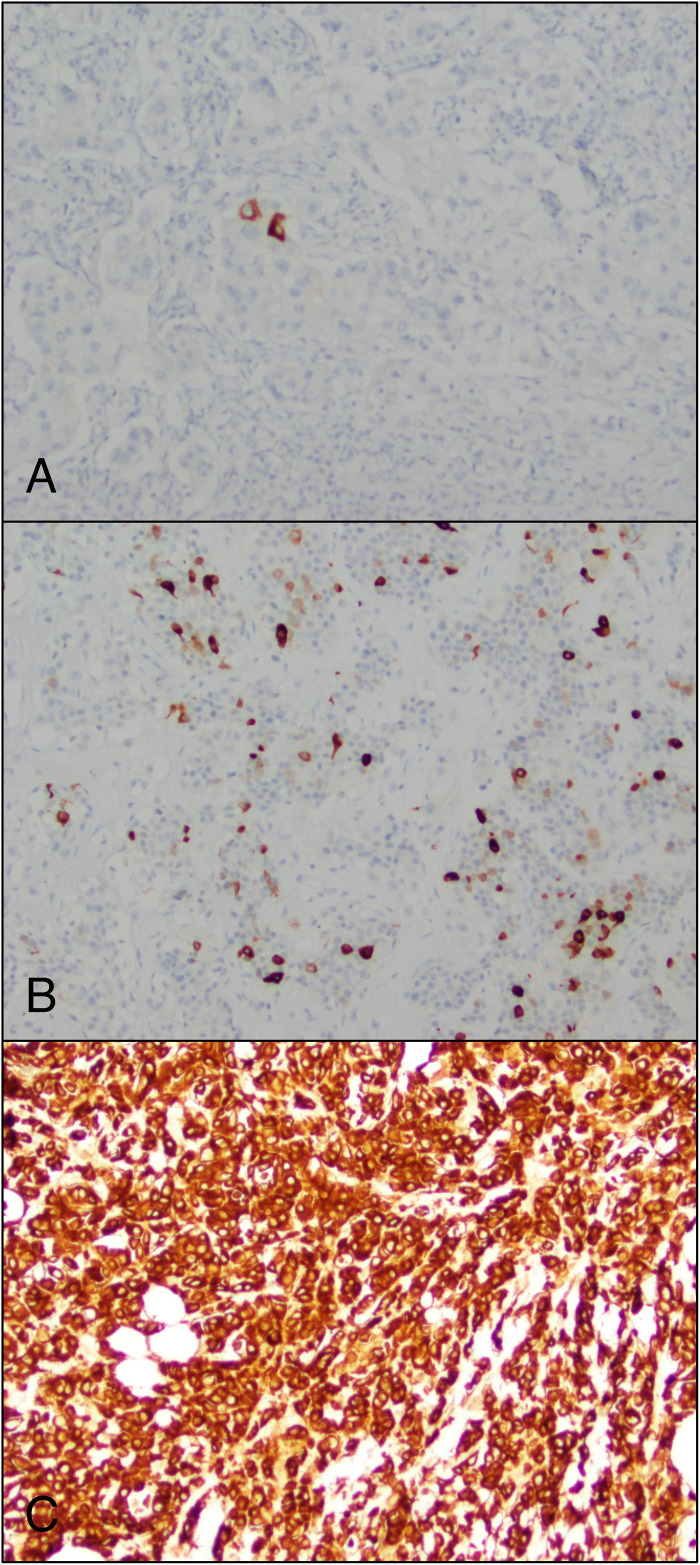
Spectrum of mammaglobin expression in triple negative breast cancer. (A) Single rare tumor cell positivity was the most common pattern of expression. (B) Focal positivity (less than 50% of tumor cells). (C) Diffuse strong positivity was rare, identified in less than 5% of all tumors (original magnification 20× [A through C]).

**Figure 4.**
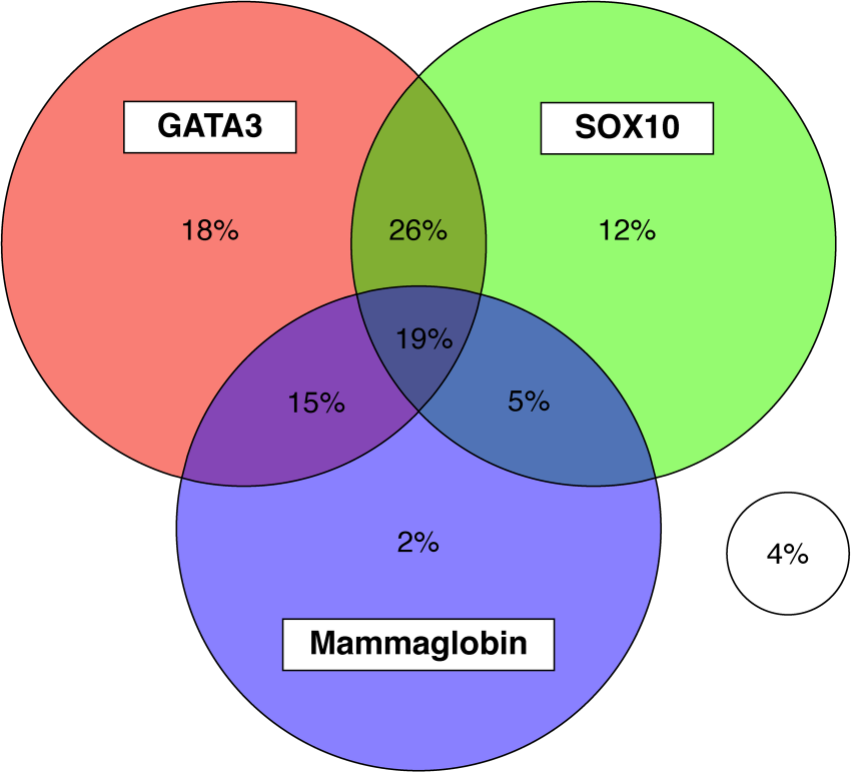
Expression of GATA3, mammaglobin, and SOX10 in triple negative breast cancer.

GATA3 was positive in 78% of tumors (489 of 629), with diffuse positivity in 36% of tumors (227 of 629). Mammaglobin was expressed in 40% of tumors (253 of 633); in more than half of these (147 of 253, 58%), positivity was limited to scattered single tumor cells. Strong and diffuse staining with mammaglobin was rare, occurring in only 5% of all tumors (Figure 3). A small proportion of tumors (6%, 39 of 623) demonstrated positivity for mammaglobin and negativity for GATA3; the majority of them were categorized as invasive breast carcinomas of no special type (82%, 32 of 39). SOX10 was positive in 62% of tumors (387 of 629); 17% of them (104 of 626) were negative for GATA3. Of the SOX10 positive tumors, 5 were also positive for reactivity with HMB45, albeit with weak intensity; none of these tumors were positive for MelanA. Of note, only 1 of the 5 tumors with positivity for SOX10 and weak reactivity with HMB45 represented invasive breast carcinoma of no special type, with the remainder representing special histologic types (3 adenoid cystic carcinomas and 1 metaplastic carcinoma). A schematic representation of the expression of GATA3, mammaglobin, and SOX10 in our cohort is presented in Figure 4.

P53 demonstrated an abnormal immunophenotype in the majority of tumors (78%, 489 of 627) with overexpression in 32% (199 of 627), null phenotype in 43% (270 of 627), and aberrant cytoplasmic staining in 3% of tumors (20 of 627). P16 was overexpressed in approximately half of the tumors (51%, 321 of 628).

### Metastatic TNBC

With respect to metastatic TNBC, almost all tumors (30 of 32) in our cohort were categorized as invasive breast carcinomas of no special type. The majority of tumors were positive for GATA3 (88%, 28 of 32) and mammaglobin (69%, 22 of 32), with frequent single cell positivity for mammaglobin (54%, 12 of 22). Approximately half of the tumors (53%, 17 of 32) were positive for SOX10. Overall, only 1 metastatic TNBC lacked expression of GATA3, mammaglobin, and SOX10.

### Triple Negative Breast Cancer Subtypes

#### Invasive Breast Carcinoma of No Special Type

The majority of tumors were categorized as invasive breast carcinomas of no special type (79%, 499 of 633). GATA3 was expressed in 77% (383 of 495); mammaglobin in 41% (203 of 491), typically with single cell positivity (24%, 116 of 491); and SOX10 was expressed in 69% of tumors (341 of 495). Overall, only 3% (14 of 495) lacked expression of GATA3, mammaglobin, and SOX10, all representing primary tumors. Of these tumors, 2 were also negative for K7.

#### Metaplastic Carcinoma

A summary of metaplastic carcinoma subtypes and pertinent IHC findings are presented in Table 3. A total of 53 metaplastic carcinomas were included in this study. Of these, 89% (47 of 53) demonstrated positivity for pankeratin and 75% (40 of 53) for K7. GATA3 was expressed in 62% (33 of 53) of tumors. SOX10 was expressed in 53% of tumors (28 of 53). Only 28% of tumors (15 of 53) were positive for mammaglobin. Approximately half of the tumors (26 of 53) demonstrated positivity for p63. Vimentin was expressed in 96% (51/53); CD34 was not expressed in any tumors. E-cadherin demonstrated aberrant staining in 30% (16/53) of tumors. Overall, almost all tumors (94%, 49 of 53) expressed at least 1 of the following markers: pankeratin, GATA3, or SOX10. The remaining 4 tumors represented the spindle cell subtype of metaplastic carcinoma.

**Table 3.** Immunohistochemical features of metaplastic carcinomas.

Type	*N*	Pankeratin	HMWK	K5	K7	P63	GATA3	MGB	SOX10	E-cadherin loss	SATB2
Matrix producing	24	23/24	18/24	17/24	22/24	6/24	13/24	9/24 (6 focal)	22/24	5/24	2/24
Squamous cell carcinoma	12	12/12	12/12	11/11	11/12	8/12	9/12	4/12 (3 focal)	2/12	0/12	0/12
Spindle cell carcinoma	7	4/7	3/7	3/7	3/7	3/7	5/7	0/7	0/7	6/7	2/7
Low-grade adenosquamous	3	3/3	3/3	3/3	3/3	3/3	3/3	2/3 (one focal)	3/3	0/3	0/3
Mixed	7	5/7	5/7	6/7	1/7	6/7	3/7	0/7	1/7	5/7	2/7

Abbreviations: MGB: mammaglobin.

#### Carcinoma With Apocrine Differentiation

**Figure 5.**
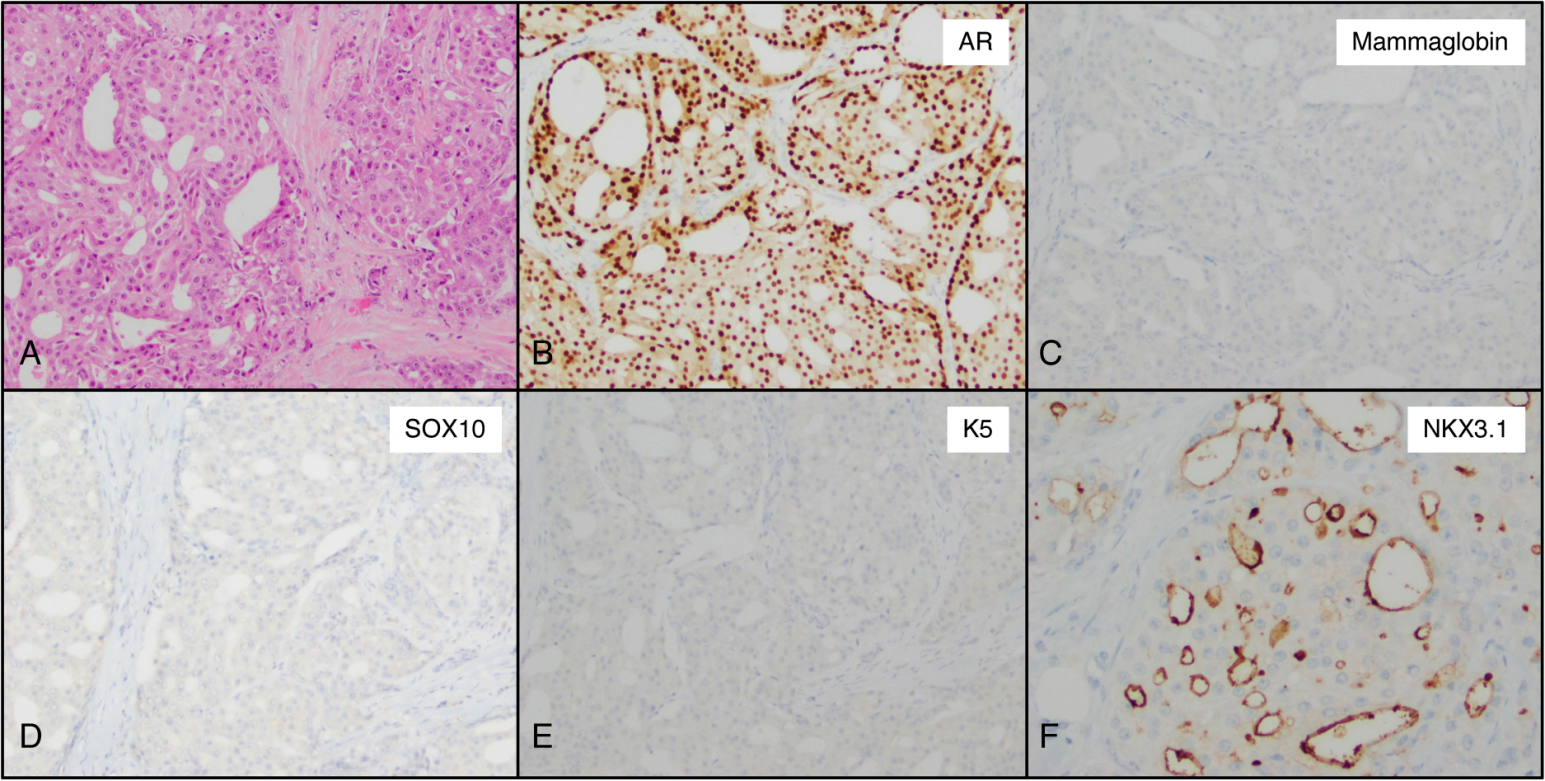
Immunohistochemical profile of triple negative breast cancer of apocrine subtype. (A) Apocrine carcinoma, hematoxylin–eosin (H&E). (B) Androgen receptor, diffuse expression. (C) Mammaglobin, no staining. (D) SOX10, no staining. (E) K5, no staining. (F) NKX3.1, luminal staining (original magnification 20× [A through F]).

Our cohort included 50 apocrine carcinomas. By definition, all tumors demonstrated positive staining with AR. The vast majority of them were positive for GATA3 (96%, 48 of 50); mammaglobin was expressed in 42% of tumors (21 of 50). In addition, 98% of tumors (49 of 50) demonstrated a lack of staining for SOX10, with the remaining tumor demonstrating focal positivity for SOX10 in less than 10% of cells; and 94% (47 of 50) demonstrated no or only focal staining for K5. NKX3.1 expression was observed in nearly half of the tumors (44%, 22 of 50), frequently with a luminal pattern of expression (Figure 5). Overall, 92% (46 of 50) of TNBC-apocrine demonstrated the AR+/SOX10−/K5(−/focal) immunophenotype.

#### Adenoid Cystic Carcinoma

Thirteen adenoid cystic carcinomas, consisting of 4 conventional and 9 basaloid tumors, were included in the study. These tumors were characterized by positivity for SOX10 (12/13) and MYB (10/13). Notably, 16 non-adenoid cystic TNBCs demonstrated variable expression of MYB (all of them in less than 25% of tumor cells); the majority of these were categorized as invasive breast carcinomas of no special type (88%, 14 of 16).

#### Invasive Lobular Carcinoma

A total of 12 invasive lobular carcinomas were identified. Of these, 9 were subclassified as apocrine lobular carcinoma. All were positive for GATA3. Mammoglobin was expressed in 2 tumors (16%) and SOX10 was expressed in 1 tumor (8%). Ten of the 12 tumors demonstrated complete loss of e-cadherin staining; 1 demonstrated partial loss and 1 demonstrated retained staining. Beta-catenin staining was lost in 8 of the 12 tumors and partially lost in 1 tumor. The single tumor with intact e-cadherin and beta-catenin expression demonstrated marked cell dyshesion, exclusive single file cell growth, and signet-ring cells.

#### Secretory Carcinoma

There were 4 secretory carcinomas included in the study. All 4 demonstrated diffuse strong positivity for mammaglobin, GATA3, and S100, as well as expression of vimentin in more than 25% of tumor cells. Three of the 4 tumors were diffusely and strongly positive for K5; the remaining tumor showed focal strong staining. Three of the 4 tumors were positive for SOX10. Two tumors demonstrated focal strong expression of androgen receptor.

#### Microglandular Adenosis-Associated Carcinoma

Our study included 5 invasive breast carcinomas of no special type and 1 matrix-producing metaplastic carcinoma arising in association with microglandular adenosis. These tumors were diffusely and strongly positive for SOX10, with variable expression of mammaglobin (2/5 tumors), GATA3 (4/5), AR (3/5), and vimentin (3/5).

#### Other Special Carcinoma Types

The remainder of cancers included 1 invasive papillary carcinoma and 1 high grade carcinoma with mucinous features. Both were positive for GATA3 and K7, and negative for TTF1, Napsin A, and CDX2.

### Immunohistochemical Clones

**Figure 6.**
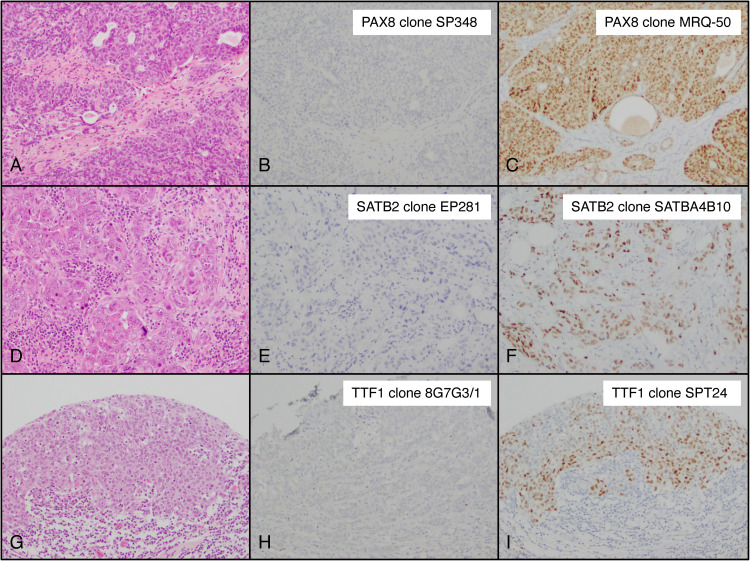
Comparison of antibody clones for PAX8, SATB2, and TTF1 in 3 representative tumors. (A) Tumor 1, hematoxylin–eosin (H&E)). (B). Tumor 1, PAX8 clone SP348. (C) Tumor 1, PAX8 clone MRQ-50. (D) Tumor 2, hematoxylin–eosin (H&E). (E) Tumor 2, SATB2 clone EP281. (F) Tumor 2, SATB2 clone SATBA4B10. (G) Tumor 3, hematoxylin–eosin (H&E). (H) Tumor 3, TTF1 clone 8G7G3/1. (I) Tumor 3, TTF1 clone SPT24 (original magnification 20× [A through I]).

Our study included multiple clones for 3 IHC markers (Table 2). Overall, clones TTF1 (8G7G3/1), PAX8 (SP348), and SATB2 (EP281) were superior to their counterparts in ruling out mammary origin (Figure 6). In particular PAX8 clone SP348 was negative in all tumors. Notably, the 3 tumors that demonstrated positivity for TTF1 (8G7G3/1) had neuroendocrine features by morphology, with 2 tumors additionally demonstrating staining for synaptophysin and/or chromogranin. In only 1 of them, TTF1 expression was strong and diffuse; this patient had no other oncologic history and the tumor was diagnosed as small cell carcinoma of the breast.

### Breast-Specific Immunohistochemical Marker Panel

Overall, 96% (607 of 629) of our cohort showed expression of GATA3, mammaglobin and/or SOX10 and only 22 tumors overall (4%) lacked expression of all these 3 markers.

**Figure 7.**
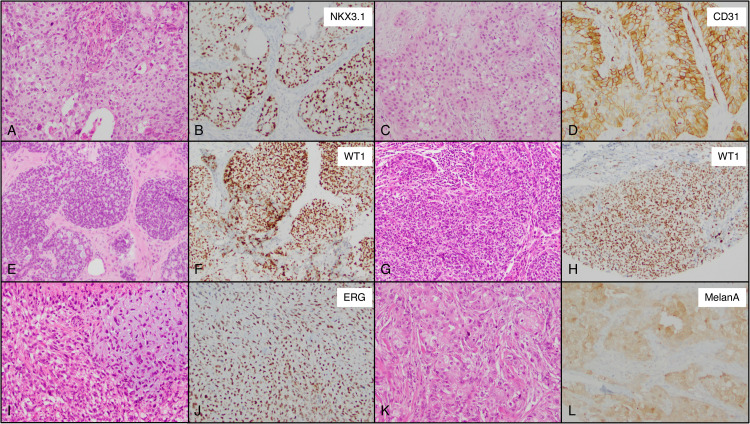
Examples of unusual marker expression in triple negative breast cancer. (A and B) Invasive breast carcinoma of no special type: H&E and corresponding NKX3.1 with diffuse expression. (C and D) Invasive breast carcinoma of no special type: H&E and corresponding CD31 with diffuse expression. (E and F) Adenoid cystic carcinoma (conventional): H&E and corresponding WT1with diffuse expression. (G and H) Invasive breast carcinoma of no special type: H&E and corresponding WT1 with diffuse expression. (I and J) Invasive breast carcinoma with metaplastic features: H&E and corresponding ERG with diffuse expression. (K and L) Invasive breast carcinoma of no special type: H&E and corresponding MelanA with moderate expression (original magnification 20× [A through L]).

None of the tumors in our cohort demonstrated expression of Napsin A, CD34 or PAX8 (clone SP348). In addition, less than 5% of tumors demonstrated positivity for ALK, CD31, chromogranin, ERG, HMB45, MelanA, PAX8 (clone MRQ-50), podoplanin (D240), SATB2 (clone EP281), TTF1 (clones 8G7G3/1, SPT24), or WT1. Similarly, ATRX, BAP1, and SMAD4 were lost in less than 5% of the total tumors. Selected examples of unusual site- or disease-specific marker expression identified in the study are presented in Figure 7.

## Discussion

TNBC represents an important diagnostic consideration in the context of carcinoma of unknown primary. However, the lack of expression for hormone receptors and many breast-specific IHC markers makes this distinction challenging. Our study examines the expression of a comprehensive panel of breast-specific and other site-specific IHC markers in a large cohort of primary and metastatic TNBC.

Previous studies have documented the utility of breast-specific IHC panels in TNBC. In a series of 57 metastatic TNBCs, Tozbikian and colleagues demonstrated that 95% of tumors were positive for either GATA3 or SOX10.^19^ In a study involving 207 primary TNBCs, the combination of SOX10, GATA3 and GCDFP-15 identified 88.4% of the tumors; the addition of AR and mammaglobin increased the yield to 93.7%.^14^ A recent study involving TMAs of 15 TNBC brain metastases demonstrated that a panel consisting of K7, GATA3 and SOX10 was able to successfully identify 100% of the tumors.^20^ Of the 32 TNBC metastases in our cohort, only 1 was negative for this panel. In our cohort of 633 TNBC tumors, a panel consisting of GATA3, mammaglobin and SOX10 similarly identified 96% of all TNBC and 97% of invasive breast carcinomas of no special type. Based on these findings, we propose that in the setting of carcinoma of uncertain lineage, particularly in the absence of hormone receptor expression, lack of expression of these 3 markers can be used to reliably exclude mammary origin.

Of note, GATA3, SOX10, and mammaglobin are not considered to be specific for breast carcinoma. GATA3 expression has been described in various tumors including urothelial carcinoma, salivary gland tumors, skin adnexal tumors, mesothelioma, mesonephric and mesonephric-like carcinomas, and germ cell tumors.^21^ Mammaglobin expression has been reported in endometrial adenocarcinomas as well as tumors of the salivary glands and skin adnexa.^22‐24^ SOX10 is expressed in melanocytic tumors, peripheral nerve sheath tumors, and tumors of the salivary glands and skin adnexa..^13,25,26^ Thus, positivity for GATA3, mammaglobin, and SOX10 in the metastatic setting must be interpreted within the clinical context for each tumor.

Carcinoma with apocrine differentiation is characterized in the fifth edition of the WHO Classification of Breast Tumors by apocrine morphology in greater than 90% of tumor cells combined with an ER negative, PR negative, AR positive immunophenotype. The importance of distinguishing apocrine differentiation is underscored by its potentially favorable prognosis in comparison to other TNBC subtypes.^27‐29^ In the 50 apocrine carcinomas included in this cohort, 92% demonstrated an AR positive, K5 negative or focal, SOX10 negative immunophenotype. This panel can be used in the differential diagnosis of apocrine and non-apocrine TNBC and pending further investigation has the potential for use in the distinction of mammary and non-mammary apocrine carcinoma.

Our study further characterizes the immunophenotypes of additional TNBC special types. Excluding the spindle cell subtype, all metaplastic carcinomas in this cohort were positive for either pankeratin, GATA3 or SOX10. Therefore, negativity for all 3 markers excludes metaplastic carcinoma in the appropriate morphologic context. Adenoid cystic carcinoma was characterized by positivity for SOX10 and MYB, as previously described.^30‐32^ The majority of invasive lobular carcinoma in this cohort demonstrated apocrine histology and were characterized by loss of e-cadherin and beta-catenin staining. Secretory carcinoma was characterized by positivity for mammaglobin, GATA3, S100 and K5. Carcinoma arising in the context of microglandular adenosis demonstrated diffuse strong positivity for SOX10 and variable expression of S100.

Our study also demonstrated p16 overexpression in approximately half of the tumors, in line with previous characterizations in TNBC cohorts.^33‐37^ P53 expression, typically defined as any staining above either 1% or 10%, has been shown in 44–71% of tumors among TNBC cohorts.^33,38‐40^ Using the p53 IHC interpretation guidelines outlined by Kang and colleagues in their evaluation of gynecologic tumors, our study identified a p53 mutant immunophenotype in 78% of tumors. Further studies examining the correlation of p53 mutant immunophenotype using the current schema with TP53 mutation detection by sequencing in TNBC are required.

There have been limited studies evaluating ATRX, BAP1 or SMAD4 IHC expression in TNBC.^41,42^ In the current cohort, the loss of ATRX, BAP1 or SMAD4 expression was very rare, occurring in less than 5% of tumors for each marker. BRAF V600E mutation is also extremely rare in TNBC,^43,44^ and in our cohort was only identified in a single tumor by VE1 IHC.

Though a frequent consideration in the differential diagnosis of metastatic lesions of unknown origin, the expression of many so-called site-specific IHC markers in TNBC has not been well established in large series. Our findings suggest that ALK, CD31, CD34, chromogranin, ERG, HMB45, MelanA, Napsin A, PAX8 (clone SP348), podoplanin (D240), TTF1 (clone 8G7G3/1), SATB2 (clone EP281), and WT1 are never, or very rarely expressed in TNBC. In the appropriate clinical context, the combination of positivity for any of these markers and negativity for the GATA3, mammaglobin and SOX10 panel can be used to reliably exclude the diagnosis of TNBC. In contrast, CA9, CDX2, NKX3.1, SATB2 (clone SATBA4B10), synaptophysin, and vimentin demonstrated variable expression in our cohort. Positivity for these markers should be interpreted with caution, and only in conjunction with additional site-specific markers in the context of excluding a diagnosis of TNBC.

Prior studies have documented the differential expression of PAX8, TTF1, and SATB2 clones in breast carcinoma.^45‐51^ The expression of these markers in our cohort was variable depending on the antibody clone applied (Figure 6). The most striking difference was observed with SATB2, with clone SATBA4B10 demonstrating positivity in 17% of tumors and clone EP281 demonstrating positivity in only 3% of tumors. Furthermore, PAX8 clone SP346 was negative in our entire cohort. Our findings further support the importance of antibody clone choice in the distinction of mammary origin.

Our study strengths include the large cohort of 633 TNBC tumors, which were reviewed and reclassified by subspecialty breast pathologists according to the WHO Classification of Breast Tumours 5th edition. This study also employs a large panel of widely available IHC markers to both primary and metastatic TNBC. Selection bias related to tumor heterogeneity was mitigated by the use of triplicate TMA cores for each tumor, which were selected by subspecialty breast pathologists. Although the purpose of the study was not to examine the expression of IHC markers in the setting of carcinoma of unknown primary, we hypothesize that the results may be used in this setting. Our study included only rare metastatic TNBC tumors to the brain or lymph nodes. As this study was conducted using TMAs, only specimens with adequate tumor volume and cellularity were included. Accordingly, core biopsies, low cellularity specimens including micrometastases as well as post-neoadjuvant therapy resections with complete or near complete pathologic response were not included. Our study did not include TRPS1, a recently described IHC marker with significantly higher expression in TNBC than GATA3.^52‐54^ However, its expression in phyllodes tumors, myofibroblastoma, and other non-metaplastic spindle cell neoplasms of the breast raises uncertainty regarding its specificity for TNBC.^55^ Additional studies are required to evaluate the expression of TRPS1 in other mammary and non-mammary origin tumors, and to further elucidate the role of TRPS1 in the workup of metastatic TNBC. While few additional markers with potential use in the diagnosis of TNBC have been described, marker selection in this study was based on availability.^56^

In summary, almost all TNBC express at least 1 of the 3 markers: GATA3, mammaglobin and/or SOX10. In addition, the expression of PAX8 (clone SP348), WT1, Napsin A, or TTF1 (clone 8G7G3/1) is never or almost never present in TNBC (the latter outside of the context of neuroendocrine carcinoma). Carcinoma of apocrine differentiation consistently demonstrates an AR positive, SOX10 negative, K5 negative or focal immunophenotype. While the majority of site-specific IHC markers do not demonstrate expression in TNBC, CA9, CDX2, NKX3.1, SATB2 (clone SATBA4B10), synaptophysin and vimentin are variably expressed and should only be used in conjunction with other markers in excluding the diagnosis of TNBC. Further, antibody clones of PAX8, SATB2, and TTF1 demonstrate differing levels of expression in TNBC; knowledge of the clone in use at each laboratory is required when interpreting these markers.
